# Effect of the CRAC Peptide, VLNYYVW, on mPTP Opening in Rat Brain and Liver Mitochondria

**DOI:** 10.3390/ijms17122096

**Published:** 2016-12-13

**Authors:** Tamara Azarashvili, Olga Krestinina, Yulia Baburina, Irina Odinokova, Vladimir Akatov, Igor Beletsky, John Lemasters, Vassilios Papadopoulos

**Affiliations:** 1 Institute of Theoretical and Experimental Biophysics, Russian Academy of Sciences, Institutskaya Str., Pushchino, Moscow Region 142290, Russia; krestinina@rambler.ru (O.K.); byul@rambler.ru (Y.B.); odinokova@rambler.ru (I.O.); akatov.vladimir@gmail.com (V.A.); ipbeletsky@gmail.com (I.B.); 2 Departments of Drug Discovery & Biomedical Sciences and Biochemistry & Molecular Biology, Medical University of South Carolina, DD504 Drug Discovery Bldg., 70 President St., MSC 140, Charleston, SC 29425, USA; JJLemasters@musc.edu; 3 The Research Institute of the McGill University Health Center, and Departments of Medicine, Biochemistry, Pharmacology and Therapeutics, McGill University, 2155 Guy Street, Montreal, QC H3H 2R9, Canada; vassilios.papadopoulos@mcgill.ca

**Keywords:** mitochondria, translocator protein (TSPO), cholesterol recognition/interaction amino acid consensus (CRAC), permeability transition

## Abstract

The translocator protein (TSPO; 18 kDa) is a high-affinity cholesterol-binding protein located in the outer membrane of mitochondria. A domain in the C-terminus of TSPO was characterized as the cholesterol recognition/interaction amino acid consensus (CRAC). The ability of the CRAC domain to bind to cholesterol led us to hypothesize that this peptide may participate in the regulation of mitochondrial membrane permeability. Herein, we report the effect of the synthetic CRAC peptide, VLNYYVW, on mitochondrial permeability transition pore (mPTP) opening. It was found that the CRAC peptide alone prevents the mPTP from opening, as well as the release of apoptotic factors (cytochrome c, AIF, and EndoG) in rat brain mitochondria (RBM). Co-incubation of CRAC, together with the TSPO drug ligand, PK 11195, resulted in the acceleration of mPTP opening and in the increase of apoptotic factor release. VLNYYVW did not induce swelling in rat liver mitochondria (RLM). 3,17,19-androsten-5-triol (19-Atriol; an inhibitor of the cholesterol-binding activity of the CRAC peptide) alone and in combination with the peptide was able to stimulate RLM swelling, which was Ca^2+^- and CsA-sensitive. Additionally, a combination of 19-Atriol with 100 nM PK 11195 or with 100 µM PK 11195 displayed the opposite effect: namely, the addition of 19-Atriol with 100 µM PK 11195 in a suspension of RLM suppressed the Ca^2+^-induced swelling of RLM by 40%, while the presence of 100 nM PK 11195 with 19-Atriol enhanced the swelling of RLM by 60%. Taken together, these data suggest the participation of the TSPO’s CRAC domain in the regulation of permeability transition.

## 1. Introduction

Cholesterol, a constituent of biological membranes, regulates the physical states of membrane phospholipid bilayers and membrane fluidity, and it determines membrane permeability. It is not uniformly distributed in cell membranes, as the highest concentration is demonstrated in the plasma membrane, whereas mitochondria have the lowest concentrations [1]. Proteins play an important role in cholesterol distribution. There are segments of integral membrane proteins that are located at the membrane interface and facilitate interactions with cholesterol-binding proteins or that have partitioned into cholesterol-rich domains, characterized by the presence of a “cholesterol recognition amino acid consensus” sequence, otherwise known as the CRAC motif (CRAC domain). A CRAC motif is defined as a sequence pattern, -L/V-(X)(1–5)-Y-(X)_(1–5)-_R/K-, in which (-X-)_(1–5)_ represents between one and five residues of any amino acid [2,3,4]. Nuclear magnetic resonance (NMR) spectroscopy of the CRAC motif has demonstrated that the side chains of the motif generate a groove that is capable of accommodating a cholesterol molecule, with the central tyrosine playing a critical role in cholesterol binding [3].

The first protein studied with a CRAC motif was the peripheral-type benzodiazepine receptor, now known as the translocator protein (TSPO) [4]. TSPO is an 18 kDa hydrophobic protein and a tryptophan-rich sensory protein oxygen sensor; it is defined as a multi-spanning membrane protein consisting of five transmembrane alpha-helices [5], and it is an extra-mitochondrial C-terminal containing a cholesterol-binding domain [3], an intra-mitochondrial N-terminal, two extra-mitochondrial loops, and two intra-mitochondrial loops. The CRAC domain of the TSPO is located at the C-terminus of the protein. TSPO transfers cholesterol (its endogenous ligand) across the membrane [6,7]. In this process, the CRAC domain is critical for cholesterol binding. Additionally, TSPO binds with high affinity to a variety of distinct chemical drugs (synthetic exogenous ligands), including the isoquinoline carboxamide, PK 11195, and others [7]. TSPO is found in all examined tissues and it is involved in various cell functions, such as steroidogenesis, cell proliferation, mitochondrial respiration, and apoptosis [8,9]. In mammalian cells, TSPO is primarily located in the outer mitochondrial membrane and it is concentrated at the outer–inner membrane contact sites. Deletion of the C-terminus of recombinant mammalian TSPO, severely reduces cholesterol uptake, although PK 11195 binding is retained [10], suggesting the existence of distinct drug ligand- and cholesterol-binding sites in TSPO [11,12,13].

Mitochondrial membrane cholesterol is known to affect the permeability of the outer mitochondrial membrane. In this relation, it is worth noting that the major outer membrane protein, VDAC (which determines outer membrane permeability [14,15]) also binds cholesterol and is probably involved in its distribution between the inner and outer membranes of the mitochondria [16]. TSPO co-localizes with VDAC [17]. This tight physical interaction between both proteins indicates that TSPO may possibly regulate VDAC function, particularly, modulating cholesterol binding to VDAC. Besides, adenine nucleotide carrier (ANT) was found to participate in the distribution of cholesterol. It should be reminded that both VDAC and ANT are considered as the main regulators of the function of the permeability transition pore.

The CRAC-like motif was determined in the BAX sequence. BAX is a pro-apoptotic member of the Bcl-2 protein family that resides in an inactive state in the cytoplasm of normal cells. Oligomer BAX forms pores in the outer mitochondrial membrane [18]. Cholesterol modulates oligomerization and the insertion of BAX into the membranes. The existence of the CRAC domain in BAX may promote the protein incorporation into the cholesterol-enriched membrane [19]. The CRAC motif is also found in transmembrane domains of connexin43 (LLIQWYIY), which is present in mitochondria, where it might interact with TSPO [20]. Interestingly, proteins shown to bind cholesterol, e.g., VDAC and TSPO, or having the CRAC motif, e.g., TSPO and Bax, participate in the regulation of permeability of the outer mitochondrial membrane.

CRAC peptides have been synthesized and examined in artificial bilayer lipid membranes (BLM). Epand et al showed that the cholesterol-binding peptide, LWYIK, was able to stimulate the formation of cholesterol-rich domains in BLM, which is composed of phosphatidylcholine and cholesterol [21]. The CRAC motif is a primary structure pattern used to identify regions that may be responsible for preferential cholesterol binding in many proteins [22]. However, at the moment nothing is known about the effect of the CRAC peptide on mitochondrial function and in particular on the initiation of the mitochondrial permeability transition pore (mPTP) opening.

To understand the mechanism of action of the CRAC peptide in the cells, a novel ligand, 3,17,19-androsten-5-triol (19-Atriol), was recently identified, which has the ability to inhibit cholesterol binding at the CRAC motif [23]. 19-Atriol binds to a synthetic CRAC peptide and inhibits steroidogenesis in MA-10 mouse Leydig tumor cells, as well as in R2C rat Leydig tumor cells, at low micromolar concentrations. In addition, 19-Atriol suppresses PK 11195-stimulated steroidogenesis, with activity in the high nanomolar range. The binding of 19-Atriol to the CRAC domain did not perturb PK 11195 binding to TSPO. TSPO has been implicated in mitochondrial permeability transition and in cell death [23,24], so 19-Atriol was supposed to facilitate cell death through CRAC domain binding [25]. Collectively, the data described above suggest that the CRAC domain is critical for the TSPO function in cholesterol transport, and 19-Atriol might operate at the level of mitochondrial cholesterol transfer. At the moment, the effect of 19-Atriol on mitochondrial function has not been examined. Therefore, taking into consideration that the initiation of mPTP opening is considered as an initial stage of programmed cell death, the aim of the present work was to examine the effect of a synthetic CRAC peptide (VLNYYVW), 19-Atriol, as well as their combined effect on mPTP opening in purified rat brain non-synaptic mitochondria (RBM) and in rat liver mitochondria (RLM). Both RBM and RLM preparations were used to compare and contrast to previous work we performed on mPTP induction by TSPO drug ligands [20,23].

## 2. Results

### 2.1. Effect of the CRAC Peptide (VLNYYVW) and the Combined Effect of the CRAC Peptide with PK 11195 on Calcium Capacity in RBM

The effect of the synthetic peptide-a fragment of the TSPO cholesterol-binding consensus, VLNYYVW, on the mPTP function of rat non-synaptic brain mitochondria was examined. The functional state of mitochondria was determined by simultaneous measurement of the membrane potential (Δψ) with a TPP^+^-selective electrode, and Ca^2+^ release was measured with a Ca^2+^ electrode. Calcium pulses (50 µM each) were added to the mitochondria to reach a threshold calcium concentration for mPTP opening. The mitochondria maintained a high Δ*Ψ*_m_ level in the presence of succinate and rotenone.

**Figure 1:**
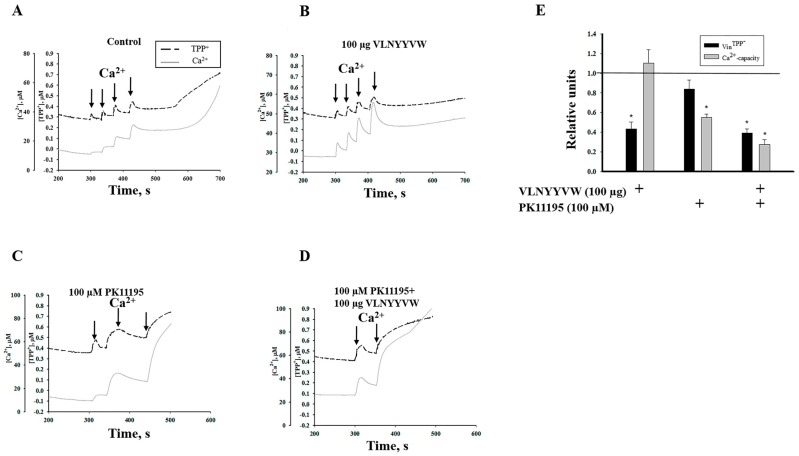
Effect of the VLNYYVW peptide and the translocator protein (TSPO) ligand, PK 11195, on Ca^2+^-induced mPTP opening in RBM (rat brain non-synaptic mitochondria). The arrows indicate where CaCl_2_ (50 µM) was added to the mitochondrial suspension. RBM were incubated in standard medium, as described in Materials and Methods. A value of *p* < 0.05 was considered to be significant (asterisk indicates *p* < 0.05). (**A**) Control RBM; (**B**) VLNYYVW (100 µg)-treated RBM; (**C**) PK 11195 (100 µM)-treated RBM; (**D**) The combined effect of VLNYYVW and PK 11195; (**E**) The quantitative characteristics of mitochondrial parameters. Ca^2+^ capacity and the rate of TPP+ influx, calculated as described in [23].

As shown in Figure 1A, the first three pulses of Ca^2+^ (added to RBM) induced a decrease in Δ*Ψ*_m_ following its restoration. At the same time, Ca^2+^ rapidly accumulated in the mitochondrial matrix until it reached 150 µM; however, the fourth addition of calcium (reaching a total of 200 µM Ca2^+^) caused Ca^2+^ release within approximately four minutes (250 s) after the last calcium addition, indicating that pore opening had initiated. Next, we checked whether a peptide with a sequence analogous to the cholesterol-binding consensus of TSPO (CRAC peptide, VLNYYVW) had the ability to change the threshold Ca^2+^ load needed for mPTP opening. The addition of 100 µg of VLNYYVW alone does not induce the opening of mPTP. Furthermore, pore opening was not observed after the fourth calcium pulses to the mitochondrial suspension (Figure 1B).

In this case, the mitochondria were more resistant, since calcium release and membrane depolarization were not observed. Then, we tested whether PK 11195, which is known to be among the most specific drugs binding to TSPO, can alter the effect of added VLNYYVW. Earlier, we reported that 100 nM PK 11195 was able to suppress mPTP opening in calcium-overloaded mitochondria, while 100 µM PK 11195 stimulated it [23]. Therefore, we used 100 µM PK 11195 to test the combined effect of PK11195 with VLNYYVW. Figure 1C shows that, in the presence of 100 µM PK 11195 alone in RBM suspension, the third addition of calcium immediately initiated pore opening. Thus, 150 µM Ca^2+^ was enough to initiate mPTP opening in the presence of 100 µM PK 11195. Next, we tested whether the VLNYYVW peptide was able to decrease the stimulating effects of PK 11195. In Figure 1D, the combined effect of 100 µg VLNYYVW and 100 µM PK 11195 on initiating of mPTP opening in RBM is shown. It was found that, taken together, these compounds significantly stimulate calcium release after two calcium additions (100 µM Ca^2+^), demonstrating that 100 µg VLNYYVW in combination with 100 µM PK 11195 is able to decrease the threshold calcium concentration by two times and accelerate pore opening. It was observed that CsA (mPTP blocker) was able to prevent acceleration of mPTP opening caused by VLNYYVW and 100 µM PK 11195 (data not shown). Figure 1E presents the comparative summary data regarding the effect of VLNYYVW and PK 11195 on calcium capacity and membrane potential under mPTP opening. In the presence of 100 µM PK 11195, the calcium capacity in calcium-overloaded RBM decreased by two times. However, PK 11195, when added to the mitochondrial suspension in combination with VLNYYVW, results in a reduction in RBM’s calcium capacity by almost three times. Thus, the VLNYYVW peptide (100 µg) alone was able to delay mPTP opening, while the peptide combined with PK 11195 strengthened the effect of the drug. These data allowed us to postulate that cooperation of the CRAC peptide with PK 11195 might decrease calcium retention and accelerate mPTP opening.

### 2.2. Effect of 19-Atriol (an Inhibitor of Cholesterol-Binding TSPO) on the Swelling of RLM

**Figure 2:**
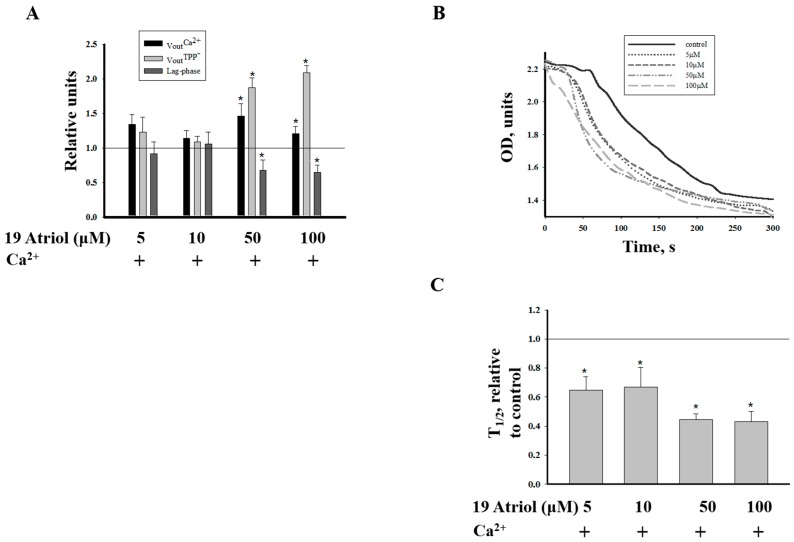
Concentration dependence of 19-Atriol on the parameters of RLM (rat liver mitochondria) functions. (**A**) Quantitative characteristics of parameters of RLM functions; (**B**) The high-amplitude swelling of RLM in the presence of different concentrations of 19-Atriol; (**C**) The average results of the half-time (T_1/2_) to reach the maximal swelling of RLM. Lines in (**A**) and (**C**) show swelling of RLM under control conditions. This parameter was calculated as described in [26]. A value of *p* < 0.05 was considered to be significant (asterisk indicates *p* < 0.05).

A novel ligand, 19-Atriol, was recently identified; it inhibits cholesterol binding at the TSPO CRAC motif, which is responsible for binding cholesterol and facilitating its translocation from the outer to inner mitochondrial membrane [25]. We used 19-Atriol to determine the possible participation of 19-Atriol in mPTP and its relationship with the CRAC peptide, VLNYYVW. Since Ca^2+^-induced swelling is a parameter of mPTP function, we examined the effect of 19-Atriol on mitochondrial swelling by measuring this swelling as a decrease of absorbance at 540 nm. For that, we used RLM, which swell better, and isolation of mitochondria from the liver allowed us to obtain a sufficient amount of mitochondria for testing RLM swelling under different conditions and for the detection of the membrane potential and Ca^2+^-induced Ca^2+^ release from RLM. First, we tested the effect of different concentrations of 19-Atriol in the range of 5–100 µM (5, 10, 50, and 100 µM) on various mPTP parameters, such as the Ca^2+^ release rate, membrane depolarization, and the time of calcium retention before pore opening (the lag-phase period). These parameters were measured in a chamber with installed selective electrodes, as in Figure 1. It was found that only 50 and 100 µM 19-Atriol had an effect on pore opening; they increased calcium release and depolarization, and shortened the lag-time period prior to calcium release, thus inducing pore opening (Figure 2A).

To further assess the effects of 19-Atriol on mPTP, we examined the effect of different concentrations of 19-Atriol on Ca^2+^-induced swelling of RLM (Figure 2B). Swelling was initiated by the addition of 200 µM Ca^2+^ to RLM (at a protein concentration of 0.5 mg/mL) incubated in standard medium (see Materials and Methods). Ca^2+^-induced swelling of RLM was initiated in the presence of 5, 10, 50, and 100 µM 19-Atriol. Ca^2+^-induced swelling of RLM (shown in Figure 2B) was accelerated by 5 and 10 µM 19-Atriol, and the effect was more pronounced in the presence of 50 and 100 µM of 19-Atriol. The diagram in Figure 2C shows the average results for the half-time (T_1/2_) required to reach 50% of the maximal swelling of RLM. The parameter, T_1/2_, strongly decreased in the presence of 50 μM and 100 µM of 19-Atriol, resulting in a two-fold reduction of the T_1/2_ parameter when compared to the control (Ca^2+^-induced swelling). Next, we examined the effect of 19-Atriol in cooperation with CRAC and with PK 11195 on Ca^2+^ accumulation and Ca^2+^-induced mPTP opening in RLM.

**Figure 3:**
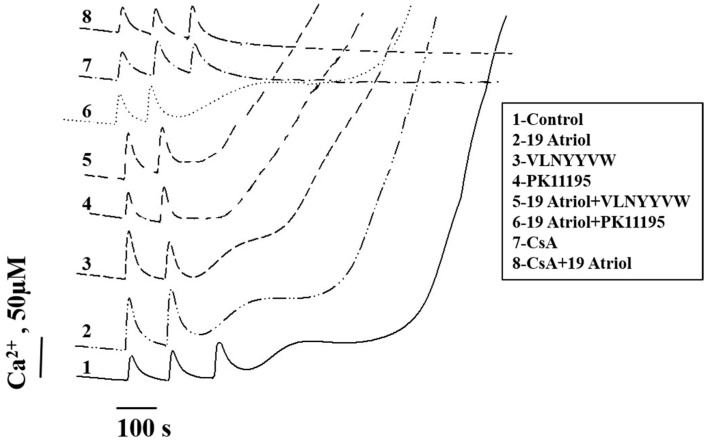
Effect of 19-Atriol, VLNYYVW, and PK 11195, as well as 19-Atriol in combination with VLNYYVW and PK 11195, on Ca^2+^ accumulation and Ca^2+^ release in RLM. Traces 7 and 8 indicate Ca^2+^ accumulation and release in the presence of CsA (an mPTP blocker).

Figure 3 demonstrates the effect of VLNYYVW and PK 11195 on the calcium capacity and calcium retention in 19-Atriol-treated RLM.

Figure 3 shows that in control RLM, pore opening was induced after the addition of the third calcium pulse (150 M Ca^2+^ in sum; curve 1) and following a lag-phase of 200 seconds. 19-Atriol was able to initiate mPTP opening at a lower calcium concentration (following the addition of 100 µM calcium), demonstrating a decrease in the threshold calcium concentration by 30% in the presence of 50 µM 19-Atriol, as compared with the control. The lag-phase was found to be 130 s (curve 2).

Curves 3–6 in Figure 3 show that 100 µM Ca^2+^ was enough to induce mPTP opening in RLM although its ability to retain calcium was rather different. Curve 3 in Figure 3 demonstrates that Ca^2+^ release was stimulated in the presence of the VLNYYVW peptide only and the lag-phase period was decreased by 30% (100 s). The effect of the CRAC peptide on mPTP opening in RLM was found to be the opposite of that seen with RBM. The presence of 100 µM PK 11195 alone caused acceleration of mPTP opening, shortening the lag-phase by 50%, compared to the 19-Atriol effect only (curve 4). Induction of mPTP opening in RLM in the presence of 19-Atriol with PK 11195, as well as in the presence of the combination of 19-Atriol and the VLNYYVW peptide is shown in curves 5 and 6. It was observed, that calcium retention (the lag-time period before pore opening) was decreased in the presence of 19-Atriol with VLNYYVW in comparison with the CRAC peptide alone, while in the presence of 19-Atriol with PK 11195 the lag-phase was prolonged by 30% in comparison with the presence of 19-Atriol alone. Since shortening of the lag-phase means accelerating mPTP opening, the results indicate that the VLNYYVW peptide itself, and combination of the peptide with 19-Atriol, significantly stimulates mPTP opening in RLM.

19-Atriol, in combination with 100 µM PK 11195, slows down calcium release and prevents pore opening, demonstrating the opposite effect, particularly since 19-Atriol, or 100 µM PK 11195 alone, facilitates mPTP opening. Because the definition of the classical mPTP is that of a Ca^2+^-induced and CsA–sensitive pore, we used a specific mPTP opening blocker to confirm the relationship of the effects reported above to mPTP. Curves 7 and 8 demonstrate the blocking of mPTP opening by CsA in control calcium-overloaded RLM, as well as in 19-Atriol-treated RLM. The same results have been obtained in experiments in the presence of CsA, performed under all conditions shown in curves 3–6. Calcium release was not found under all these conditions tested (not shown). The results obtained demonstrate that mPTP opening, induced in RLM by 19-Atriol and by 19-Atriol in combination with the CRAC peptide, were Ca^2+^- and CsA-sensitive, suggesting the potential participation of the CRAC domain in mPTP function.

### 2.3. Combined Effect of 19-Atriol and the CRAC Peptide on Ca^2+^-Induced RLM Swelling

**Figure 4:**
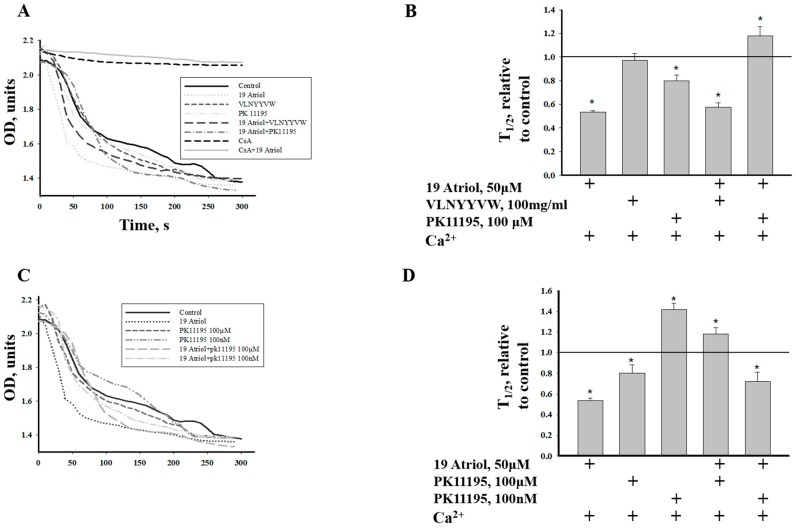
The effect of 19-Atriol, VLNYYVW, PK 11195, and the combined effect of 19-Atriol with VLNYYVW and PK 11195 on RLM swelling. A value of *p* < 0.05 was considered to be significant (asterisk indicates *p* < 0.05). (**A**) Curves of RLM swelling in the presence of 19-Atriol, VLNYYVW, and PK 11195 (100 µM). Trace 1 indicates the absence of swelling without Ca^2+^ addition. Trace 8 demonstrates the absence of swelling in the presence of CsA; (**C**) Curves of RLM swelling in the presence of 19-Atriol, as well as in the presence of 19-Atriol in combination with different concentrations of PK 11195 (100 µM or 100 nM); (**B**,**D**) Average results of the half-time (T_1/2)_ swelling, in comparison with the control swelling of RLM in the presence of Ca^2+^ without any other additions. Lines in (**B**) and (**D**) show swelling of RLM under control conditions.

We examined the combined effect of 19-Atriol with the CRAC peptide on Ca^2+^-induced RLM swelling (as an additional parameter of mPTP function) to obtain additional evidence on the relationships between the effects of the drugs on mPTP. Figure 4A shows that Ca^2+^-induced RLM swelling is significantly accelerated in the presence of 19-Atriol (trace 2), and it was completely prevented in the presence of CsA (trace 8). A decrease in RLM swelling was found in the presence of the CRAC peptide, VLNYYVW (trace 3), however, RLM swelling in the presence of both 19-Atriol and VLNYYVW was stimulated.

The diagram in Figure 4B shows the average results for the half-time (T_1/2_) when 50% of the maximal RLM swelling was reached. The given data demonstrate that VLNYYVW does not induce RLM swelling, while the inhibitor, 19-Atriol alone and in combination with the CRAC peptide, is able to enhance Ca^2+^-induced swelling by two times. CsA prevented RLM swelling, supporting the notion that the effect of 19-Atriol is related to mPTP function. Strong protection of RLM swelling was found in the presence of 19-Atriol and 100 µM PK 11195, when swelling was diminished by 30% in comparison with the control. This was an unexpected result, particularly since 100 µM PK 11195 alone stimulates mPTP opening in both RLM and RBM, while 100 nM PK11195 is able to prevent pore opening. Therefore, we also compared the combined effect of 100 nM PK 11195 and 19-Atriol, and we subsequently compared this with the effect of 100 µM PK 11195 together with 19-Atriol on Ca^2+^-induced RLM swelling. Figure 4C,D show that 100 nm PK 11195 alone suppresses RLM swelling, while 100 µM PK 11195 initiates it. However, the combination of 19-Atriol with 100 nM PK 11195 or with 100 µM PK 11195 displays the opposite effect: namely, the addition of 19-Atriol and 100 µM PK 11195 into an RLM suspension suppresses Ca^2+^-induced RLM swelling by 40%, while the presence of 100 nM PK 11195 with 19-Atriol enhanced RLM swelling by 60%. Thus, the CRAC peptide itself does not influence RLM swelling, but the inhibitor of the CRAC domain (19-Atriol), which has a blocking effect on the CRAC peptide, is able to stimulate Ca^2+^-induced RLM swelling. The results support the hypothesis suggesting that the CRAC domain is involved in mPTP function or regulation. The effect of 100 nM PK 11195 in combination with 19-Atriol indicates that effect of 19-Atriol is linked to TSPO, as only TSPO has nanomolar affinity to PK 11195.

### 2.4. Effect of the CRAC Peptide (VLNYYVW) on the Release of Apoptotic Factors (Cytochrome c, AIF, and EndoG) from RBM under mPTP Opening

**Figure 5:**
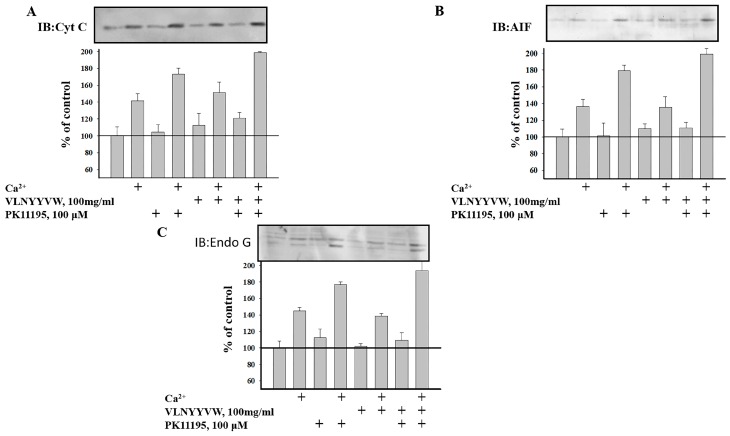
Release of pro-apoptotic factors cytochrome c, AIF, and EndoG from non-synaptic RBM under mPTP opening. (**A**)-Cytochrome c release; (**B**) AIF release; (**C**) Endo G release. Probes were taken in control conditions without added Ca^2+^ and in Ca^2+^-overloaded mitochondria, as well as in the absence/presence of VLNYYVW and PK 111195, or in the combination of VLNYYVW with PK 111195. Western blots of the supernatant fraction of non-synaptic mitochondria were obtained following centrifugation. Proteins of the supernatant fractions were separated in SDS-PAGE after Western blot.

It is known that the induction of mPTP initiates the release of apoptotic factors, such as cytochrome c, apoptosis-inducing factor (AIF), and endonuclease G (EndoG), from mitochondria. Earlier, we observed that in Ca^2+^-overloaded RBM, the release of apoptotic factors (cytochrome c, AIF, and EndoG) was increased [27]. Therefore, we compare herein the levels of cytochrome c, AIF, and EndoG under a control condition and after mPTP induction. We tested the release of these factors in supernatants of RBM treated with the VLNYYVW peptide. The aliquots of non-synaptic RBM were taken from the chamber under the examined conditions, and the aquilots were subsequently centrifuged. The samples were used for electrophoresis with following Western blot. Identification of cytochrome c, AIF, and Endo G were performed using the monoclonal anti-cytochrome c antibody, the polyclonal anti-AIF antibody and the polyclonal anti-EndoG antibody (see Materials and Methods). Under the control conditions, when mPTP is closed, the release of cytochrome c was not observed in the RBM supernatant. The level of cytochrome c increased insignificantly under the same conditions in the presence of VLNYYVW alone, or in the presence of VLNYYVW together with 100 µM PK 11195 (Figure 5A).

When the pore is closed, the same effect was found for AIF release, but the level of AIF release was weaker when compared with that of the cytochrome c release (Figure 5B). Furthermore, the release of EndoG was found not to be changed in the presence of VLNYYVW. The induction of mPTP leads to cytochrome c release from RBM. The presence of VLNYYVW does not lead to cytochrome c release, whereas 100 µM PK 11195 enhances the release of cytochrome c; however, the strongest release (by nearly two times) was observed in the presence of VLNYYVW together with 100 µM PK 11195 (Figure 5A). AIF (Figure 5B) and EndoG (Figure 5C) release were stimulated by calcium, when mPTP opened. The CRAC peptide was not able to further accelerate mPTP opening, but the CRAC peptide was able to strengthen AIF and EndoG release in cooperation with PK 11195 (Figure 5B,C). In sum, the results give reason to suppose that there is possible modulation of the VLNYYVW effect by PK 11195, indicating that their combined effect might be involved in the initiation of apoptosis, as well as in the release of apoptotic factors.

## 3. Discussion

The structure and activity of a membrane protein is modulated by both the interaction with its ligands and with the lipid membrane environment in which it resides. Certain drugs may modulate function by inducing physical changes in the membrane environment and by affecting the conformation and function of proteins within a membrane [28,29] that might lead to changing of the membrane permeability. The formation of cholesterol-rich domains might be used as an example. Some proteins are known to sequester to cholesterol-rich domains (raft domains) forming a CRAC motif [3,4,30,31,32]. In this work, for the first time, we examine the effect of the synthetic peptide-a fragment of the TSPO cholesterol-binding consensus, VLNYYVW, on induction of the mPTP, which is considered as the initial stage of apoptosis.

The initiation of apoptosis is usually preceded by the loss of the mitochondrial membrane potential (Δ*Ψ*_m_), high-amplitude swelling of the mitochondria, and apoptotic factor release, which are found during mPTP opening. Until now, the exact composition of the pore complex has not been established, but it is clear that increased permeability of the inner membrane also depends on the permeability of the outer mitochondrial membrane, mainly on VDAC [14]. The role of the C-terminal end (CRAC motif) of TSPO in mPTP has not been examined until now, therefore, the effect of the CRAC peptide (VLNYYVW) on the induction of mPTP opening in mitochondria was investigated. Here, it was shown that VLNYYVW alone does not induce the opening of mPTP in RBM (Figure 1B). However, VLNYYVW in combination with PK 11195 (100 µM) was able to accelerate pore opening in a CsA-sensitive manner. Taken together, these compounds seem to significantly stimulate calcium release from RBM, thus decreasing the threshold calcium concentration by two times and accelerating pore opening (Figure 1D). These data allowed us to hypothesize that, in the presence of PK 11195, which can modify physical properties of the membrane BLM [33,34], the CRAC peptide might promote the permeability transition. Interestingly, the peptide, VLNYYVW, was found to be able to stimulate mPTP opening in RLM (Figure 3, curve 3). In the presence of the CRAC peptide, the calcium threshold concentration needed for pore opening was decreased by 30%. That could be due to the tissue-dependent lipid composition of phospholipids, forming a mitochondrial phospholipid bilayer.

Recently, a CRAC domain ligand (19-Atriol) was identified; it is capable of inhibiting cholesterol binding at the TSPO CRAC motif [25]. In the present studies, for the first time, we used 19-Atriol to examine whether there is a relationship between 19-Atriol and mPTP. At an effective concentration of 100 µM, 19-Atriol was found to induce mPTP opening, increase calcium release and membrane depolarization, and shorten the time period prior to calcium release. Also, 100 µM of 19-Atriol caused high-amplitude RLM swelling, which was found to be Ca^2+^-induced and CsA-sensitive, This finding suggests that there is a cause–effect relationship between 19-Atriol action and mPTP function. The experiments performed herein also revealed that the CRAC peptide itself can initiate mPTP opening in RLM. The combination of 19-Atriol with the VLNYYVW peptide caused further reduction of calcium retention in RLM (the lag-time before pore opening) by three-fold, leading to the acceleration of mPTP opening (Figure 3, curves 2, 3, and 5). 19-Atriol was shown to bind to a synthetic CRAC peptide [25], and it also inhibited binding of cholesterol at the CRAC domain in TSPO. Taken together, these results suggest that blocking of the CRAC domain, and thus suppression of cholesterol binding, might stimulate mPTP opening. The effect of 19-Atriol in combination with 100 µM of PK 11195 was found to exert an opposite effect. In this case, there was a prolongation of calcium retention by 50%, slowing down the pore opening. Thus, it is likely that PK 11195 is able to stabilize the CRAC-containing C-terminal end of TSPO, since PK 11195 is able to overcome the 19-Atriol-dependent acceleration of mPTP opening (Figure 3, curves 2 and 6). 19-Atriol-dependent mPTP opening was prevented by CsA, highlighting the relationship between the effect of 19-Atriol and mPTP function.

Additional evidence supporting this hypothesis was obtained while examining the effect of 19-Atriol in cooperation with the CRAC peptide and PK 11195 on high-amplitude Ca^2+^-induced RLM swelling. 19-Atriol-treated RLM swelling was increased by 50% when compared with the control (Figure 4A,B). Combination of 19-Atriol with the VLNYYVW peptide was able to prevent swelling of RLM, compared to 19-Atriol-dependent swelling. The swelling of RLM, stimulated by 100 µM PK 11195, demonstrates an opposite effect in cooperation with 19-Atriol. Indeed, when together, they were able to prevent the swelling of RLM (Figure 4A,B). Additionally, the 19-Atriol-stimulated Ca^2+^-induced swelling of RLM was CsA-sensitive. RLM swelling was caused by 19-Atriol in combination with 100 µM PK 11195. Swelling of RLM in the presence of CsA was not observed with all probes tested. These data support the relationship between 19-Atriol-dependent swelling of RLM and mPTP function. Earlier, we showed that 10–100 nM PK 11195 prevents mPTP opening [34]. If the addition of 19-Atriol with 100 µM PK 11195 results in the suppression of Ca^2+^-induced swelling by 40%, then a combination of 100 nM PK 11195 with 19-Atriol induces RLM swelling by 60%. Given that nanomolar concentrations of PK 11195 bind specifically with TSPO, the effect of 100 nM PK 11195 with 19-Atriol seems to be TSPO-dependent and related to mPTP. It should be noted that this effect is reminiscent of the strong stimulation of mPTP opening in RBM, as a result of the combined effect of 100 nM PK 11195 with G3139 (a blocker of VDAC channels) [35]. VDAC regulates the flux of calcium ions. The VDAC channel shows very low permeability to Ca^2+^ in its normal open state. However, after VDAC closure, the permeability to Ca^2+^ can be increased by 10 times. Ca^2+^ flux into mitochondria through VDAC can lead to calcium accumulation and induction of mPTP opening. G3139 closes the VDAC channel that lead to increase of calcium permeability. Earlier, we reported that cooperation of PK 11195 together with G3139 resulted in VDAC closure and a subsequent acceleration of mPTP opening [35]. We propose that 19-Atriol, probably in complex with 100 nM PK 11195, acts in a manner that is similar to the G3139–PK 11195 combination. Since VDAC binds cholesterol, it could be a target for 19-Atriol action, which might close the VDAC channel. Blocking of cholesterol binding may lead to an increase in membrane permeability, which is sensitive to CsA. Interestingly, cholesterol is able to bind to CsA with low affinity [36]. Kinnunen et al. showed that the penetration of CsA into the lipid seems to be a specific lipid–drug interaction, which could be involved in the change of the conformation and/or orientation of CsA. The conformation/orientation of CsA in the membrane is probably sensitive to cholesterol [36]. Thus, the interaction between mPTP players and cholesterol in the mitochondrial membranes of BLM might be involved in the regulation of mPTP function. As proteins containing CRAC domains (BAX, VDAC, connexin43) were found in lipid rafts, it is possible to suppose that raft structures participate in mPTP formation/regulation. The mitochondrial cholesterol pool seems to be an important factor in the regulation of mitochondrial membrane permeabilization and cell death. It has been reported that TSPO and its ligands are implicated in mitochondrial permeability transition during apoptosis [23,37,38]. mPTP is a main checkpoint of programmed cell death in brain cells, and the induction of mPTP initiates the release of apoptotic factors, such as cytochrome c, AIF, and EndoG from mitochondria. Cytochrome c does not release from calcium-overloaded RBM in the presence of VLNYYVW. However, the CRAC peptide in combination with PK 11195 was able to stimulate cytochrome c release by two-fold as the result of this cooperation (Figure 5A). VLNYYVW alone was not able to stimulate AIF and EndoG release in calcium-overloaded RBM; however, in cooperation with PK 11195, the release of AIF and EndoG (Figure 5B,C) was accelerated. The results obtained provide reason to assume that a possible cooperative interaction between VLNYYVW and PK 11195 exists, which might lead to the release of apoptotic factors, as well as to the initiation of apoptosis. Earlier, it was shown that PK 11195-dependent induction of mPTP opening in RBM was CsA-sensitive. We hypothesize that the VLNYYVW+PK11195-dependent activation of apoptotic factors released from RBM is related to mPTP function.

Taken together, these results demonstrate that the CRAC peptide and its ligand, 19-Atriol, might mediate mPTP function and apoptosis. This effect might be modulated by PK 11195. PK 11195, incorporated into the lipid chains, is able to alter membrane fluidity, much in the same way as cholesterol [39]. Miccoli et al. [40] found an increase in mitochondrial membrane fluidity following exposure to 10 nM PK 11195 for 24 h, which was regulated via binding to TSPO. By taking these data into consideration, we suggest that PK 11195, the CRAC peptide, and 19-Atriol react with the outer membrane. If PK 111195 is able to influence mitochondrial membrane properties [39,40], then it might promote the interaction of VDAC with TSPO, both of which are mPTP regulators. Cholesterol is a critical membrane component. Many of cholesterol’s effects are due to the accumulation of cholesterol in the mitochondrial membranes. Targeting mitochondrial cholesterol in a number of pathologies, such as steatohepatitis, neurodegenerative diseases, or cancer, has been reported [41,42]. The ability of the CRAC domain to bind to cholesterol allowed Lecanu et al. to show that another CRAC peptide (VLNYYVWR) had a direct action on plaque, as CRAC enabled the removal of cholesterol from plaque depot sites [43]. The authors found that the administration of the VLNYYVWR human CRAC sequence to guinea pigs fed with a high-cholesterol diet resulted in reduced circulating cholesterol levels. The CRAC peptide thus appears to be a safe prototypical drug for the treatment of dyslipidemia and atherosclerosis. Their results thus indicate that the CRAC peptide might constitute a novel and safe treatment for hypercholesterolemia and atherosclerosis [43]. However, new additional investigations should be undertaken to further understand the multifaceted function of TSPO, as well as CRAC peptides.

## 4. Materials and Methods

### 4.1. Animals

Brain and liver mitochondria were isolated from the total brain of male Wistar rats. All experiments were performed in accordance with the “Regulations for Studies with Experimental Animals” (Decree of the Russian Ministry of Health of 12 August 1997, No. 755). The protocol was approved by the Commission on Biological Safety and Ethics of the Institute of Theoretical and Experimental Biophysics, Russian Academy of Science (November 2014, protocol N45).

### 4.2. Isolation of Rat Liver Mitochondria (RLM)

Mitochondria were isolated from rats using a standard method [44] featuring a homogenization medium containing 210 mM mannitol, 70 mM sucrose, 1 mM EGTA, 0.05% bovine serum albumin (BSA) fraction V, and 10 mM Tris (pH 7.3). The homogenate was centrifuged at 800× *g* for 10 min to pellet nuclei and damaged cells. The supernatant, which contained the mitochondria, was centrifuged for 10 min at 9000× *g*. Sedimented mitochondria were washed twice in medium containing EGTA and BSA for 10 min at 9000× *g* and resuspended in the same medium. The protein concentration was determined using the Bradford assay.

### 4.3. Isolation of Rat Brain Mitochondria (RBM)

The rats were fasted overnight before decapitation and isolation of the mitochondria. The brain was rapidly removed (within 30 s) and placed in an ice-cold solution containing 0.32 M sucrose, 1 mM EDTA, 0.5% BSA (fraction V), and 10 mM Tris-HCl (pH 7.4). All solutions used were ice-cold; all manipulations were carried out at +4 °C. The tissue was homogenized in a glass homogenizer; the ratio of brain tissue to isolation medium was 1:10 (*w*/*v*). The homogenate was centrifuged at 2000× *g* for 3 min. A mitochondrial pellet was obtained after centrifugation of the 2000× *g* supernatant at 13,500× *g* for 10 min. RBM was suspended in ice-cold solution containing 0.32 M sucrose, 0.1 mM EDTA, 0.05% BSA (fraction V), and 10 mM Tris-HCl (pH 7.4), and they were washed by centrifugation at 12,500× g for 10 min. The protein concentrations in the mitochondrial suspensions were 25–30 mg/mL. For the final step, the mitochondria were purified on Percoll gradient [26].

### 4.4. Evaluation of Mitochondrial Functions

The mitochondrial membrane potential was measured as described earlier [23] by determining the distribution of tetraphenylphosphonium (TPP^+^) in the incubation medium with a TPP^+^-selective electrode and Ca^2+^ transport were determined with a Ca^2+^-sensitive electrode (Nico, Moscow, Russia). Mitochondria (1 mg protein/mL) were incubated in a medium containing 125 mM KCl, 10 mM Tris, and 2 mM K_2_HPO_4_, pH 7.4, at 25 °C. During the experiments, succinate (5 mM) was used as a substrate, and rotenone (5 µM) was added to the measuring medium to block Complex I dehydrogenases. The mPTP opening in RLM was induced by a threshold Ca^2+^ concentration (each addition of Ca^2+^ contained 50 μM). The RLM swelling was measured as a change in light scattering in a mitochondrial suspension at 540 nm (A_540_) using a Tecan I-Control infinite 200 spectrophotometer at 25 °C (Tecan Group Ltd., Männedorf, Switzerland). The standard incubation medium for the swelling assay contained 125 mM KCl, 10 mM Tris, 2 mM KH_2_PO_4_, 5 mM succinate, and 5 µM rotenone. The concentration of the mitochondrial protein in each well was 0.5 mg of protein per mL. Swelling was initiated by the addition of 200 nmol of Ca^2+^ per mg of protein. The swelling process was characterized by the time needed to reach the half-maximal light-scattering signal (T_1/2_).

### 4.5. Electrophoresis and Immunoblotting of Mitochondrial Proteins

Samples ranging from 20–30 µg of protein were mixed with Laemmli solubilization solution and boiled for 3 minutes. For immunoblotting, mitochondrial proteins solubilized in Laemmli buffer were separated under denaturing conditions on 12.5% sodium dodecyl sulfate (SDS)-polyacrylamide gel electrophoresis (PAGE) gels and transferred to nitrocellulose membranes. Precision Plus Pre-stained Standards from Bio-Rad Laboratories (Hercules, San-Diego, CA, USA) were used as markers. After overnight blocking, the membrane was incubated with the appropriate primary antibody.

### 4.6. Cytochrome c, AIF, and EndoG Release

Mitochondria (1 mg of protein per mL) were incubated in the medium containing 125 mM KCl, 10 mM Tris-HCl, 0.4 mM K_2_HPO_4_, and 5 µM rotenone, pH of 7.4, at 25 °C. Succinate (5 mM potassium succinate) was used as a mitochondrial respiratory substrate. The threshold Ca^2+^ concentration was reached by adding Ca^2+^ to the mitochondrial suspension in an open chamber with installed selective electrodes. A total of 100 nanomoles Ca^2+^ per mg of protein was sufficient to initiate mPTP opening. The release of cytochrome c, AIF, EndoG, and CNP was detected in the supernatant before and after the induction of mPTP opening. Aliquots (100 μL) were taken from the chamber and centrifuged at 10,000× *g* for 6 min. Then, samples of 30 μL of supernatant were taken and diluted in 10 μL of 4× Laemmli buffer. The pellet fraction was diluted in 90 μL of 1× Laemmli buffer. The samples were used for electrophoresis following Western blot. The monoclonal anti-cytochrome c antibody was used at 1:2000 dilution (# DLN 06724 from Dianova, Hamburg, Germany), the polyclonal anti-AIF antibody was used at 1:500 dilution (#PC 536 from Calbiochem^®^; Merck Millipore, Billerica, MA, USA), and the polyclonal anti-EndoG antibody was used at 1:500 dilution (AB3639 from Merck Millipore, Billerica, MA, USA).

### 4.7. Quantification and Statistical Analysis

Quantification of the band densities from Western blots was carried out using a GS800 calibrated densitometer and Gel Pro software. Films were scanned and band intensities were quantified. For statistical analysis, relative levels of protein density were expressed as means ± SD from at least three –four different experiments. Significance was determined by using Student’s *t*-test. A value of *p* < 0.05 was considered to be significant (asterisk indicates *p* < 0.05). ANOVA with Bonferoni post-hoc comparison was used with statistical significance at *p* < 0.05 for Figure 5.

## 5. Conclusions

In the present study we report that the peptide VLNYYVW, designed on the TSPO’s CRAC domain, prevents the mPTP from opening and the release of apoptotic factors in RBM. VLNYYVW did not induce swelling in RLM. 19-Atriol, an inhibitor of the cholesterol-binding activity of TSPO’s CRAC domain, alone and in combination with the peptide was able to stimulate RLM swelling in a Ca^2+^- and CsA-sensitive manner. The TSPO specific drug ligand PK 11195 modulates the effects of the CRAC peptide and 19-Atriol on the induction of mPTP opening and apoptotic factor release. Taken together these results suggest that TSPO via its C-terminal CRAC domain participates in mPTP function/regulation and apoptosis initiation; moreover, TSPO drug ligands are pharmacologic regulators of this process.

## Abbreviations

TSPO	Translocator Protein
CRAC	Cholesterol recognition/interaction amino acid consensus
mPTP	Mitochondrial permeability transition pore
